# Development of a Transient Wellbore Heat Transfer Model Validated with Distributed Temperature Sensing Data

**DOI:** 10.3390/s25216583

**Published:** 2025-10-26

**Authors:** Rion Nakamoto, Smith Leggett

**Affiliations:** Bob L. Herd Department of Petroleum Engineering, Texas Tech University, 807 Boston Ave, Lubbock, TX 79409, USA; sleggett@ttu.edu

**Keywords:** fiber optic sensing, distributed temperature sensing, transient heat transfer models, thermal slug velocity, circulation test, geothermal

## Abstract

Distributed temperature sensing (DTS) has long been employed in the oil and gas industry to characterize reservoirs, optimize production, and extend well life. More recently, its application has expanded to geothermal energy development, where DTS provides critical insights into transient wellbore temperature profiles and flow behavior. A comprehensive understanding of such field measurements can be achieved by systematically comparing and interpreting DTS data in conjunction with robust numerical models. However, many existing wellbore models rely on steady-state heat transfer assumptions that fail to capture transient dynamics, while fully coupled wellbore–reservoir simulations are often computationally demanding and mathematically complex. This study aims to address this gap by developing a transient wellbore heat transfer model validated with DTS data. The model was formulated using a thermal-analogy approach based on the theoretical framework of Eickmeier et al. and implemented with a finite-difference scheme. Validation was performed by comparing thermal slug velocities predicted by the model with those extracted from DTS measurements. The results demonstrated strong agreement between modeled and measured slug velocities, confirming the model’s reliability. In addition, the modeled thermal slug velocity was lower than the corresponding fluid velocity, indicating that thermal front propagates more slowly than the fluid front. Consequently, this computationally efficient approach enhances the interpretation of DTS data and offers a practical tool for improved monitoring and management of geothermal operations.

## 1. Introduction

DTS is a fiber-optic technique that measures temperature based on Raman scattering within the fiber [1]. In this process, photons interacting with molecular vibrations are scattered in two forms: Stokes and anti-Stokes [2]. While Stokes scattering produces lower-energy photons, anti-Stokes scattering generates higher-energy photons whose intensity strongly depends on temperature [3]. By analyzing the ratio between these two signals, the temperature distribution along the fiber can be determined. Most DTS systems employ optical time-domain reflectometry (OTDR), where short light pulses are sent through the fiber and the backscattered signal is recorded [4]. The time delay of the returning signal indicates the location of the scattering event, allowing temperature to be mapped along the fiber length [1]. DTS has become an essential technology in upstream oil and gas applications, enabling real-time downhole measurement for monitoring and optimizing well performance [5]. More recently, it has drawn particular attention to geothermal energy development, where downhole temperature profiling provides critical insights into subsurface heat transfer and overall well performance.

Wellbore fluid temperature modeling has advanced significantly since the pioneering study of Ramey [6], who introduced a single-phase vertical flow model incorporating a time-dependent function and an effective heat transfer coefficient. While sufficiently accurate for long-term steady-state condition, this approach could not capture short-term transients. Subsequent studies extended Ramey’s framework to account for multiphase flow, well deviation, and variable thermal properties [7,8,9]. A major step forward came in the mid-1990s, when Hasan and co-workers proposed simplified analytical models that enabled direct calculation of circulating fluid temperatures [10,11], and later developed fully coupled wellbore–reservoir simulators capable of analyzing both pressure-transient and temperature behavior in single- and two-phase flow systems [12,13]. Collectively, these developments have greatly expanded the ability to model complex thermal and flow processes across a wide range of wellbore and reservoir conditions.

Despite these advances, existing wellbore heat transfer models still face key limitations. Many rely on steady-state thermal assumptions and therefore cannot adequately represent transient temperature responses, while fully coupled mass–momentum–energy simulators, although comprehensive, are mathematically demanding and computationally intensive. Previous studies have also examined transient responses from field observations. For example, one study interpreted the propagation of thermal slugs detected by fiber-optic systems in the Utah FORGE 16B producer. Apparently, they considered the thermal slug velocities as equal to the fluid velocities in the wellbore [14]. This interpretation has offered a practical basis for flow profiling in fractured wells and has been applied in geothermal developments [15,16,17,18], particularly in Enhanced Geothermal Systems (EGS) [14,19]. However, this assumption inherently oversimplifies the underlying heat transfer processes, highlighting the need for a more rigorous understanding of thermal conduction and propagation in relation to fluid velocity.

To overcome the above limitations, this study employs a thermal-analogy approach for modeling transient wellbore heat transfer, drawing on the methodology proposed by Eickmeier et al. [20]. This approach provides three key advantages. First, it enables reliable prediction of temperature changes under both steady and transient conditions, during both heating and cooling phases. Second, by treating fluid flow in the wellbore as steady, it avoids solving transient mass and momentum equations, while still capturing the essential unsteady thermal behavior. Third, it naturally accounts for heat conduction into the surrounding formation without requiring a reservoir flow model, thereby reducing computational effort.

Accordingly, this study has three main objectives: (i) to develop a transient wellbore heat transfer model capable of accurately predicting temperature profiles with a relatively simple mathematical approach, (ii) to validate the model against field observations by comparing temperature profiles and thermal slug velocities between them, and (iii) to enhance the understanding of transient temperature behavior by investigating and comparing fluid and thermal slug velocities. The remainder of this paper is organized as follows. Section 2 details the methodology of the developed numerical model and the experimental design of the fluid circulation test, from which most of the input parameters for the numerical simulation were determined. Section 3 presents the DTS results obtained from the circulation test and provides a detailed description of the validation procedure and interpretation of the results. Finally, Section 4 concludes the study and highlights potential directions for future research.

## 2. Materials and Methods

### 2.1. Methodology for the Numerical Model

This section outlines the methodology of the developed numerical model. In addition to the basic assumptions and the governing equations derived from the theory of Eickmeier et al. [20], the model specification also includes the resistance and capacitance values required for the thermal analogy, together with the geometry ratio used to represent spatial discretization.

#### 2.1.1. Basic Assumptions

The numerical model relies on simplifying assumptions that allow for a practical representation of heat transfer without excessive computational complexity. These assumptions define the scope of the model and provide a basis for subsequent formulation of the governing equations. The key assumptions are summarized as follows.

1. Unsteady heat transfer is considered, such that temperature distribution and heat flux within the wellbore vary with time. In contrast, fluid flow is treated as steady state, based on the assumption that pressure and velocity transients propagate rapidly and can be neglected.
2. The fluid inside the wellbore is assumed to remain in the liquid phase throughout the simulation, as typical EGS operating conditions maintain pressures and temperatures that prevent vapor formation. Therefore, phase change and gas evolution are not considered.
3. Heat transfer between the wellbore fluid and the surrounding formation is assumed to occur only in the radial direction, while vertical conduction is neglected because the axial temperature gradient over each control volume is small compared to the radial gradient. Convection is included in two forms:
  - Forced convection when fluid is actively flowing, where the convective heat transfer depends on the flow rate.
  - Natural convection when the fluid is stagnant such as a tubing-casing annulus, where buoyancy-driven mixing causes heat exchange with the surroundings.
4. Thermal properties of the materials involved, including density, heat capacity, and thermal conductivity, are assumed constant for each layer, such as flowing fluid, tubing, and casing and independent of both temperature and pressure.
5. The forced convection heat transfer coefficient *h_t_*, is defined as a function of the varying flow rate, whereas the natural convection heat transfer coefficient *h_a_*, depends on temperatures of the surrounding solid layers.
6. Injection conditions, including surface injection temperature and flow rate, may be adjusted at each simulation time step.
7. The geothermal temperature profile is prescribed either as a linear function of depth or from DTS data.
8. The geometry of the wellbore, including tubing, casing, and wellbore diameters, is assumed to be constant with depth.
9. Frictional, kinetic, and thermodynamic effects are neglected because their contributions to the energy balance are negligible under EGS operation conditions. Omitting them simplifies the model without significantly affecting accuracy.
10. Heat conduction between any two elements follows Fourier’s law, whereas heat convection between elements is described by Newton’s law of cooling.

#### 2.1.2. Governing Equations

**Figure 1:**
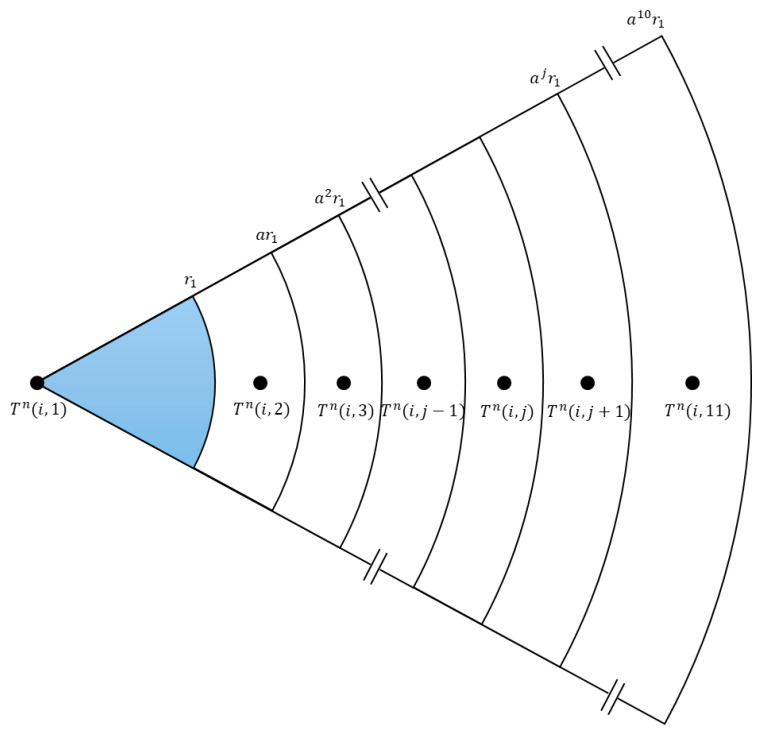
Plan view for one-dimensional radial heat transfer.

**Figure 2:**
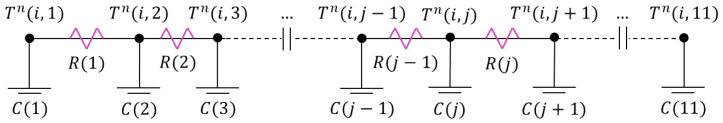
Thermal analogy for one-dimensional radial heat transfer.

**Figure 3:**
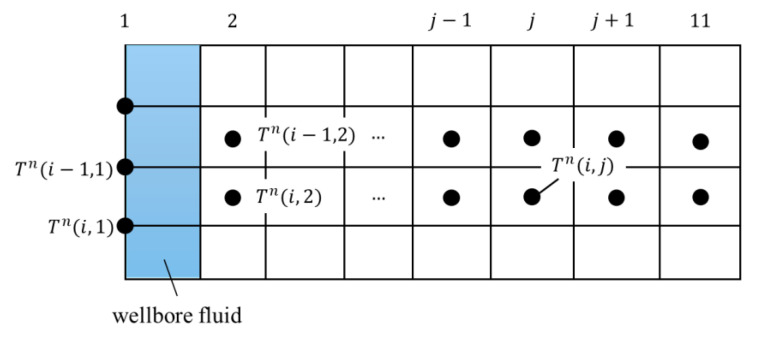
Elevation view for one-dimensional radial heat transfer.

In this study, heat transfer in the reservoir is modeled as one-dimensional radial conduction within a cylindrical system. To simplify the analysis, a unit-depth, pie-shaped sector with a central angle of one radian at vertical level *i* is considered, as shown in Figure 1. Owing to the radial symmetry of the wellbore, this small sector is representative of the entire formation. The wellbore radius is denoted as *r*_1_, and subsequent radial positions of the grid blocks are defined as in Figure 1. To avoid boundary effects, the radial grid extends outward, with the outer radius of the *j*th grid cell (*j* = 10) given by *a^j^r*_1_, where *a* is the geometric ratio and *j* = 1, 2, …, 10. This discretization scheme is flexible and can be adapted to different well configurations, including tubing, casing, and cement layers, without altering the fundamental concept. Figure 2 and Figure 3 illustrate the equivalent thermal network and elevation view corresponding to the schematic in Figure 1.

The governing equations for the wellbore system are derived by considering one-dimensional radial heat transfer. While conduction governs heat transfer in the surrounding formation grid cells, both conduction and convection must be accounted for within the flowing wellbore fluid. For clarity, the derivation is presented in two parts: the energy balance in the formation grid cell, and the energy balance in the flowing fluid. The governing equations presented in this section follow the formulation of Eickmeier et al. [20], unless otherwise stated. Additional supporting expressions are adapted from standard heat transfer theory.

##### Energy Balance in the Formation Grid Cells

The energy balance for the *j*th cell at level *i* (*j*  ≥  2) is given by:

(1) $$E_{j-1\to j}^{\mathrm{cond}}-E_{j\to j\;+\;1}^{\mathrm{cond}}\;=\;\frac{\partial U_{j}}{\partial t}$$

where $E_{j-1\to j}^{cond}$ is the energy in by conduction from *j*-1th to *j*th cell, $E_{j\to j+1}^{cond}$ is the energy out from *j*th to *j* + 1th cell, and $\partial U_{j}/\partial t$ is the change in internal energy in the *j*th cell.

Mathematically, this can be written using thermal analogy as [20]:

(2) $$\frac{T^{n}\left(i,j-1\right)-T^{n}\left(i,j\right)}{\frac{R(j-1)}{2\pi dz}}-\frac{T^{n}\left(i,j\right)-T^{n}\left(i,j+1\right)}{\frac{R(j)}{2\pi dz}}=\;\frac{C(j)\cdot 2\pi dz\left[T^{n+1}\left(i,j\right)-T^{n}\left(i,j\right)\right]}{\mathrm{dt}}$$

where $T^{n}\left(i,j\right)$ is the temperature for *j*th cell at level *i* at time step *n*. R(j) and C(j) are resistance and capacitance values for jth cell, and *dz* and *dt* represent vertical increment and time increment. Rearranging Equation (2), $T^{n+1}\left(i,j\right)$ is expressed as [20],

(3) $$T^{n+1}\left(i,j\right)=\left[1-A(j)\mathrm{dt}\right]T^{n}\left(i,j\right)+\frac{\mathrm{dt}}{C(j)}\left[\frac{T^{n}\left(i,j-1\right)}{R(j-1)}+\frac{T^{n}\left(i,j+1\right)}{R(j)}\right]$$

where

(4) $$A(j)=\frac{R(j-1)+R(j)}{R(j-1)R(j)C(j)}$$

At the closed outer boundary (*j* = 11, see Figure 1, Figure 2 and Figure 3), the boundary condition $T^{n}\left(i,j\;+\;1\right)=T^{n}\left(i,j\right)$ is applied in Equation (3),

(5) $$T^{n+1}\left(i,j\right)=\left[1-\frac{\mathrm{dt}}{C\left(j\right)R\left(j-1\right)}\right]T^{n}\left(i,j\right)+\left[\frac{\mathrm{dt}}{C\left(j\right)R\left(j-1\right)}\right]T^{n}\left(i,j-1\right)$$

As shown in Figure 3, for *j* = 2, $T^{n}\left(i,j-1\right)$ denotes the fluid temperature at level *i*. In this case, it is approximated as the average of the temperatures at levels *i-*1 and *i* as:

(6) $$T^{n}\left(i,j-1\right)=\frac{T^{n}\left(i-1,j-1\right)\;+{\;T}^{n}\left(i,j-1\right)}{2}$$

This distinction results from the fact that nodes at *j* = 1 are placed directly on the grid points, while for j ≥ 2, they are positioned at the centers of the grid cells. This condition is then applied at the inner boundary in Equation (3),

(7) $$T^{n+1}\left(i,j\right)=\left[1-A\left(j\right)\mathrm{dt}\right]T^{n}\left(i,j\right)+\frac{\mathrm{dt}}{C\left(j\right)}\left[\frac{T^{n}\left(i-1,j-1\right)+T^{n}\left(i,j-1\right)}{2R\left(j-1\right)}+\frac{T^{n}\left(i,j+1\right)}{R\left(j\right)}\right]$$

To avoid numerical instability, it is recommended that the term $\left[1-A\left(j\right)\mathrm{dt}\right]$ in Equation (3) always remain positive. This leads to the following constraint:

(8) $$\mathrm{dt}\;\leq \frac{\;R(j-1)R(j)C\left(j\right)}{R(j-1)\;+\;R(j)}$$

##### Energy Balance in the Flowing Fluid

The energy balance for the flowing fluid at level *i* (*j* = 1) is given by:

(9) $$E_{i-1}^{\mathrm{conv}}-E_{i}^{\mathrm{conv}}-E_{j\to j+1}^{\mathrm{cond}}\;=\;\frac{\partial U_{j}}{\partial t}$$

where $E_{i-1}^{\mathrm{conv}}$ is energy in by convection at *i*-1 and $E_{i}^{\mathrm{conv}}$ is energy out by convection at *i*. Mathematically, this can be written using thermal analogy as:

(10) $$q_{w}\rho_{w}c_{\mathrm{pw}}\cdot T^{n}\left(i-1,j\right)-q_{w}\rho_{w}c_{\mathrm{pw}}\cdot T^{n}\left(i,j\right)-\frac{\frac{T^{n}\left(i-1,j\right)+T^{n}\left(i,j\right)}{2}-T^{n}\left(i,j\;+\;1\right)}{\frac{R\left(j\right)}{2\pi dz}}\;=\frac{\;C\left(j\right)\left[T^{n+1}\left(i,j\right)-T^{n}\left(i,j\right)\right]}{\mathrm{dt}}$$

where $q_{w}$ is flow rate, $\rho_{w}$ is density, and $c_{\mathrm{pw}}$ is specific heat of the flowing fluid. Rearranging Equation (10), $T^{n+1}\left(i,j\right)$ is expressed as,

(11) $$T^{n+1}\left(i,j\right)=\left[\frac{\mathrm{Hdt}}{C\left(j\right)}\right]T^{n}\left(i,j+1\right)+\left[\frac{\left(2q_{w}\rho_{w}c_{\mathrm{pw}}-H\right)\mathrm{dt}}{2C\left(j\right)}\right]T^{n}\left(i-1,j\right)+\left[1-\frac{\left(2q_{w}\rho_{w}c_{\mathrm{pw}}+H\right)\mathrm{dt}}{2C\left(j\right)}\right]T^{n}\left(i,j\right)$$

where

(12) $$H=\frac{2\pi dz}{R(j)}$$

Equation (11) introduces an additional constraint to avoid numerical instability:

(13) $$1-\frac{(2q_{w}\rho_{w}c_{\mathrm{pw}}+H)\mathrm{dt}}{2C(j)}\;\geq \;0$$

which yields:

(14) $$C(j)\;\geq \;\frac{\mathrm{dt}}{2}(2q_{w}\rho_{w}c_{\mathrm{pw}}\;+\;H)$$

When the heat capacity of the flowing fluid is small, the condition above may impose an undesirably small time step. For long-term simulations, this issue can be mitigated by assigning an artificial value to *C*(*j*). Such an adjustment is acceptable, as the change in the fluid’s heat content is negligible compared with the total heat input over extended periods. In the present simulations, however, no artificial value for *C*(*j*) was introduced. An additional requirement associated with Equation (11) is given by,

(15) $$\frac{\left(2q_{w}\rho_{w}c_{\mathrm{pw}}-H\right)\mathrm{dt}\;}{2C(j)}\geq \;0$$

which yields:

(16) $$H\;\leq \;2q_{w}\rho_{w}c_{\mathrm{pw}}$$

#### 2.1.3. Resistance and Capacitance Values

**Figure 4:**
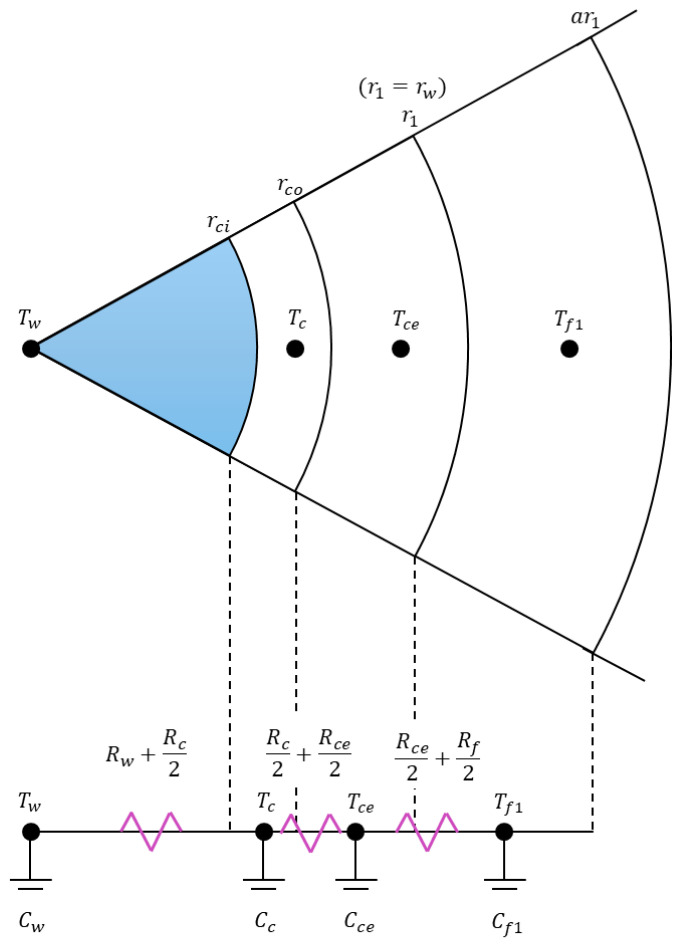
Resistance and capacitance values for a wellbore cross section.

The governing equations derived in the previous sections do not provide a direct procedure for determining thermal resistance and capacitance values. Instead, they are written in a general form that can be applied to many different well configurations. To use them in practice, however, appropriate resistance and capacitance values must be assigned to each layer of the well system. As an example, consider the case of water injection into the casing string. The corresponding well configuration is shown in Figure 4, where $r_{\mathrm{ci}}$ and $r_{\mathrm{co}}$ denote the inner and outer radii of the casing, respectively, and $r_{1}$ denotes the outer radius of the cement sheath. The rate of convective heat transfer $q_{\mathrm{conv}}$ from the casing inner wall at temperature $T_{ci}$ to the flowing fluid at temperature $T_{w}$ is governed by Newton’s law of cooling and is expressed as:

(17) $$q_{\mathrm{conv}}\;=\;2\pi r_{\mathrm{ci}}dzh_{w}(T_{ci}-T_{w})$$

In contrast, the rate of conductive heat transfer $q_{\mathrm{cond}}$ through $\;q_{\mathrm{conv}}$ the casing, from the outer wall at temperature $T_{\mathrm{co}}$ to the inner wall at temperature $T_{\mathrm{ci}}$, is governed by Fourier’s law of heat conduction and is expressed as:

(18) $$q_{\mathrm{cond}}\;=\;\frac{2\pi k_{s}dz\left(T_{ci}-T_{\mathrm{co}}\right)}{\ln\left(r_{\mathrm{co}}/r_{\mathrm{cn}}\right)}$$

Since 2πdz is already incorporated into the governing equations, the thermal resistance of each layer in this configuration is given by the following equations.

(19) $$\mathrm{Forced}\;\mathrm{Convective}\;\mathrm{Fluid}\;\mathrm{Resistance}:\;R_{w}=\frac{1}{\;h_{w}r_{\mathrm{ci}}}$$

(20) $$\mathrm{Casing}\;\mathrm{Resistance}:\;R_{c}=\frac{\ln\left(r_{\mathrm{co}}/r_{\mathrm{ci}}\right)}{k_{s}}$$

(21) $$\mathrm{Cement}\;\mathrm{Resistance}:\;R_{\mathrm{ce}}=\frac{\ln\left(r_{1}/r_{\mathrm{co}}\right)}{k_{\mathrm{ce}}}$$

(22) $$\mathrm{Formation}\;\mathrm{Resistance}:\;R_{f}=\frac{\ln\left(a\right)}{k_{f}}$$

where $R_{w}$, $R_{c}$, $R_{\mathrm{ce}}$, and $R_{f}$ are the resistance values for the flowing fluid, casing, cement, and formation. $k_{s}$, $k_{\mathrm{ce}}$, and $k_{f}$ are thermal conductivity values of the casing, cement, and formation. The forced convective heat transfer coefficient, $h_{w}$, for the flowing fluid is determined using the Dittus–Boelter correlation [21,22,23], which is described in Appendix A.1. In cases where annuli are present and the fluids within them give rise to natural convection, the corresponding heat transfer coefficient, $h_{a}$, is evaluated proposed by Dropkin and Sommerscales [24], as outlined in Appendix A.2.

With reference to thermal analogy in Figure 4, *R*(*j*) in the governing equations can be expressed as follows:

(23) $$R(1)\;={\;R}_{w}\;+\;\frac{R_{c}}{2}$$

(24) $$R(2)=\frac{R_{c}}{2}+\frac{R_{\mathrm{ce}}}{2}$$

(25) $$R(3)=\frac{R_{\mathrm{ce}}}{2}+\frac{R_{f}}{2}$$

(26) $$R(j)={\;R}_{f}$$

where *j* = 4, …, 13. It should be noted that *R*(*j*) represents only a portion of the total thermal resistance. Specifically, R(j)/2πdz corresponds to the thermal resistance, while the factor 2πdz is already incorporated into the governing equations in Equations (2) and (10).

With reference to thermal analogy in Figure 4, the thermal capacitance of each layer is given by the following equations.

(27) $$\mathrm{Forced}\;\mathrm{Convective}\;\mathrm{Fluid}\;\mathrm{Capacitance}:\;C_{w}=C\left(1\right)={\;\rho }_{w}\cdot \pi r_{1}^{2}dz\cdot c_{p_{w}}$$

(28) $$\mathrm{Casing}\;\mathrm{Capacitance}:C_{c}=C\left(2\right)=\frac{W_{c}\cdot c_{p_{c}}}{2\pi }$$

(29) $$\mathrm{Cement}\;\mathrm{Capacitance}:\;C_{\mathrm{ce}}=C\left(3\right)=\frac{\rho_{\mathrm{ce}}\cdot (r_{1}^{2}-r_{\mathrm{co}}^{2})\cdot c_{p_{\mathrm{ce}}}}{2}$$

(30) $$\mathrm{Formation}\;\mathrm{Capacitance}:\;C_{f1}=C\left(4\right)=\frac{r_{1}^{2}\left(a^{2}-1\right)\rho_{f}c_{p_{f}}}{2}\;\;C(j+1)={\;a}^{2}C(j)$$

where *j* = 4, …, 12. $C_{w}$, $C_{c}$, $C_{\mathrm{ce}}$, and $C_{f1}$ are the capacitance values, $W_{c}$ is the weight of casing, $\rho_{w}$, $\rho_{\mathrm{ce}}$, and $\rho_{f}$ are density values, and $c_{p_{w}}$, $c_{p_{c}}$, $c_{p_{ce}}$, and $c_{p_{f}}$ are specific heat values of each layer. It should be noted that *C*(*j*) in Equation (28) through Equation (30) represent only a portion of the thermal capacitance in each layer, unlike Equation (27) and consistent with the definitions in the governing equations in Equations (2) and (10).

#### 2.1.4. Geometry Ratio

The geometry ratio *a*, introduced in the previous sections as the parameter governing the radial grid spacing, should be selected such that the temperature at the outer boundary (10th grid, measured from the grid with inner radius of $r_{1}$) remains essentially unchanged throughout the simulation period. This requires choosing an appropriate dimensionless radius $r_{D}$ corresponding to the dimensionless time $t_{D}$, based on the dimensionless temperature drop function per unit heat flow, $T_{D}\left(r_{D},t_{D}\right)$. The dimensionless radius $r_{D}$ is defined as:

(31) $$r_{D}\;=\;\frac{r}{r_{w}}$$

Hence, $r_{D}$ for the configuration in Figure 4 is expressed as:

(32) $$r_{D}\;=\;\frac{a^{10}r_{1}}{r_{1}}\;=\;a^{10}$$

The dimensionless time $t_{D}$ is defined as:

(33) $$t_{D}\;=\;\frac{\alpha t}{r_{w}^{2}}$$

Thus, the appropriate value of $r_{D}$ can be determined from the specified dimensionless time $t_{D}$ and the corresponding dimensionless temperature drop function per unit heat flow, $T_{D}\left(r_{D},t_{D}\right)$. Tabulated values of this function for various $r_{D}$ and $t_{D}$ are available in the literature [25].

### 2.2. Experimental Procedures

**Figure 5:**
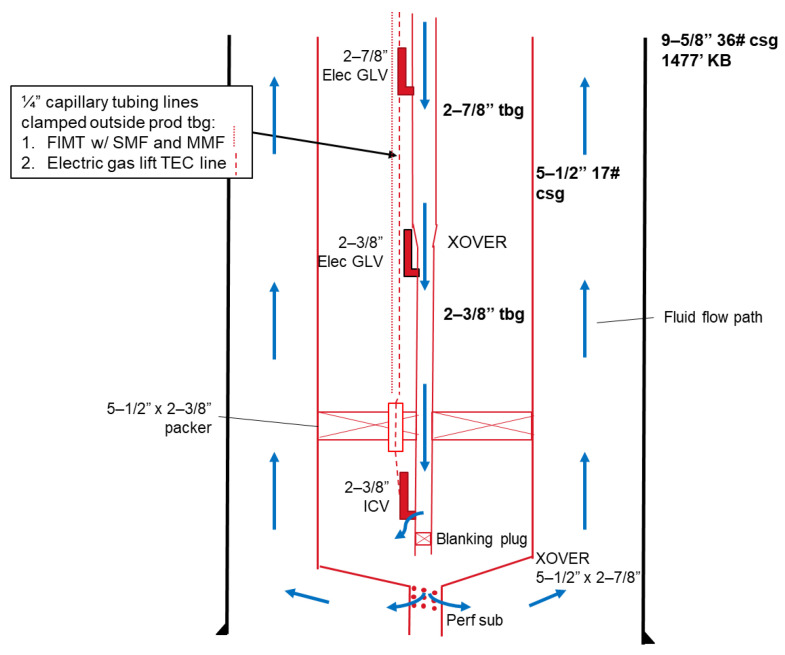
Well schematic of RR#2.

In order to test the proposed model, a circulation test was conducted on 7 March 2025, in the Red Raider #2 (RR#2) well at the Oilfield Technology Center, Texas Tech University. During the circulation test, water was injected into the well through the tubing and circulated back up through the annulus between the 5 1/2” and 9 5/8” casings. The annulus between the tubing and the inner casing was filled with nitrogen, and the annulus between the inner and outer casings was filled with water. Figure 5 presents the well schematic of RR#2. The bule lines in Figure 5 show the fluid flow path.

At the wellhead, fluid temperature was monitored using a thermocouple, and flow rate was measured with a Coriolis meter. DTS data were also acquired from a fiber optic cable clamped outside the tubing, providing spatial and temporal temperature profiles. These measurements served as inputs for the numerical model.

The operation was carried out in three stages: (1) system preparation and clean-up, (2) two hot-water injections separated by shut-in periods for thermal recovery, and (3) a final cold-water injection. All returns were collected in a frac tank. A detailed planned test schedule is provided in Table 1.

Although the circulation test followed the planned schedule outlined in Table 1, minor deviations occurred during field execution. The actual sequence of injections, shut-in periods, and monitoring activities is summarized in Table 2. These records were used to interpret the temperature responses and validate the numerical model.

### 2.3. Model Assumptions and Input Parameters

The numerical model was constructed based on the general assumptions described in Section 2.1.1. In addition to these fundamental assumptions, several test-specific considerations were incorporated. These additional considerations are summarized as follows:

(1) The tubing string consists of approximately 1337 ft of 2 7/8” tubing and 68 ft of 2 3/8” tubing. Since the majority of the tubing string consists of 2 7/8” tubing, the entire string was reasonably modeled with a uniform diameter of 2 7/8” for simplicity and consistency.
(2) Although fluid circulation could occur in the annulus space between the 5 1/2” and 9 5/8” casings, the relatively large capacity of the 9 5/8” casing makes any significant fluid movement in this region negligible. For modeling purposes, heat transfer across this annulus was therefore represented by natural convection.
(3) The simulation was designed to reproduce conditions during a 20 min time window, specifically spanning from 09:48:00 to 10:08:00, which corresponds to the duration of the test of interest.
(4) The initial temperature profiles were defined directly from DTS measurements. In particular, the initial temperature profile of the flowing fluid was determined using data recorded at 09:47:30, just before the first injection of hot water. For the remaining layers, the geothermal gradient was adopted based on earlier data collected at 09:25:36, prior to the onset of circulation, in order to represent the undisturbed formation conditions.

A detailed summary of the simulation inputs used in the first hot water injection is presented in Table 3, Table 4 and Table 5. In particular, the casing and cement thermal properties listed in Table 4 and Table 5 were determined with reference to commonly used values reported in some studies [26,27,28].

Although Table 5 does not provide separate values for the formation density $\rho_{f}$ and the specific heat $c_{f}$, the volumetric heat capacity $\rho_{f}c_{f}$ was derived through calculation using the following equation.

(34) $$\hat{\alpha }=k_{f}/\rho_{f}c_{f}$$

where $k_{f}$ is the formation thermal conductivity and $\hat{\alpha }$ is the thermal diffusivity. Using the values reported in Table 3 and Table 5, the volumetric heat capacity was calculated as $\rho_{f}c_{f}=35$, which was adopted in this study.

**Figure 6:**
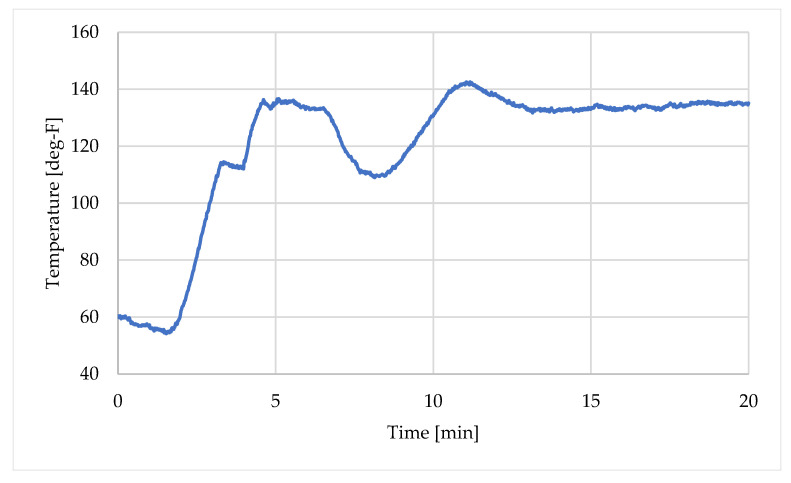
Wellhead temperature of the injected fluid measured by thermocouple.

Table 6 summarizes the temporal variation in flow rate at the wellhead, as measured by a Coriolis meter during this period. For the injection fluid, thermocouple measurements at the wellhead were used as a transient boundary condition for the injection fluid, as illustrated in Figure 6.

**Figure 7:**
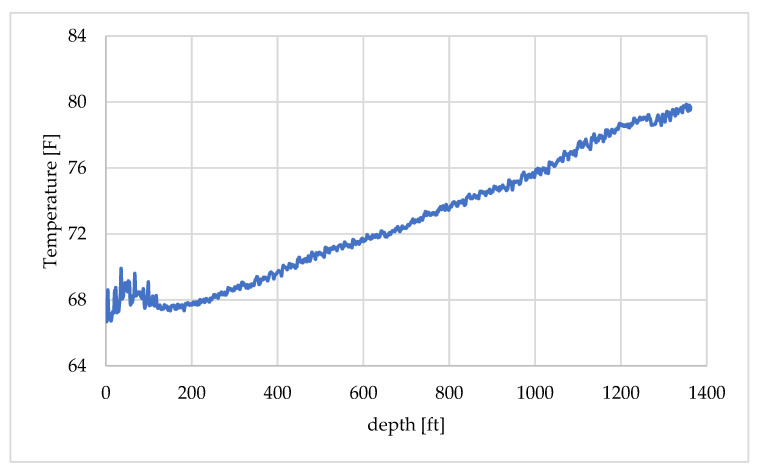
Initial temperature profile of the flowing fluid.

**Figure 8:**
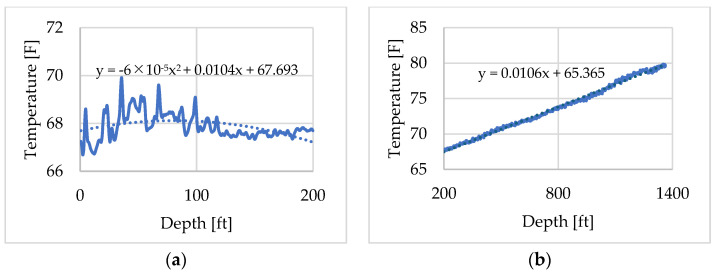
Initial temperature profile of the flowing fluid: (**a**) 0–200 ft, (**b**) 200–1400 ft.

The DTS temperature profile recorded at 09:47:30, shown in Figure 7, was selected as the initial condition in the tubing fluid. In Figure 7, depth is referenced from the wellhead, which corresponds to 0 ft. The time 09:47:30 was chosen because, although water injection had started at 09:34, it stopped after a while due to a surface pipe leak as noted in Table 2. Consequently, just before the first hot-water injection at 09:48, the DTS profile near the wellhead exhibited elevated temperatures that deviated from the normal geothermal gradient. To construct the initial temperature distribution for the numerical model of the flowing fluid, the profile was divided into two depth intervals: 0–200 ft and 200–1400 ft as shown in Figure 8a,b. Approximation curves were fitted to each interval to interpolate the temperature distribution. The spatial and temporal resolutions of the DTS measurements were 0.833 ft (0.25 m) and 30 s, respectively. Depth calibration, conducted using DTS temperature profiles obtained before and during the circulation test, indicated that the wellhead corresponded to a depth of 352.667 ft in the DTS record.

**Figure 9:**
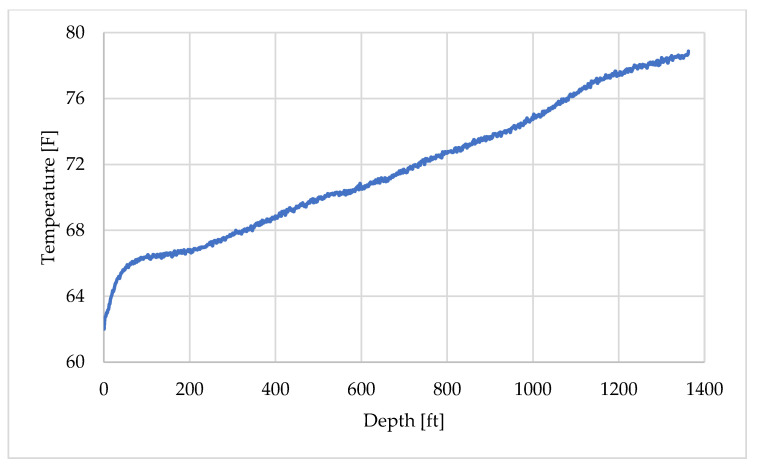
Initial temperature profile for non-fluid layers.

**Figure 10:**
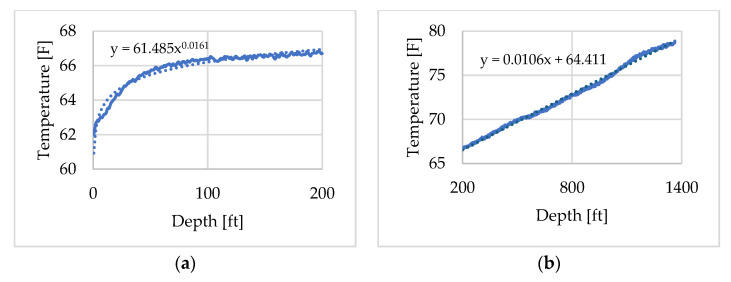
Initial temperature profile for non-fluid layers: (**a**) 0–200 ft, (**b**) 200–1400 ft.

Similarly, the DTS temperature profile recorded at 09:25:36 shown in Figure 9, was selected to represent the geothermal gradient for the other layers. This time was chosen because the record was obtained prior to 09:34 and therefore reflects the undisturbed geothermal gradient. A slight temperature drop can be observed very close to the wellhead, which is attributed to the cold weather conditions at the time of measurement, as the experiment was conducted in early March. The profile was also divided into two depth intervals and assigned using fitted approximation curves, as illustrated in Figure 10a,b.

## 3. Results and Discussion

This section presents and discusses the results of the circulation test. Section 3.1 is dedicated to the analysis of waterfall plots. It first introduces the DTS results for the entire circulation period, then highlights the waterfall plot of the initial warm-up phase, which provides the basis for later extraction of the thermal slug velocity. For comparison, a waterfall plot generated from the numerical model is also presented. Section 3.2 then focuses on the thermal slug velocity, where the extraction procedure is described in detail and applied to both the DTS data and the numerical model results, enabling a direct comparison between the two. Finally, the section concludes by examining the relationship between the fluid velocity and the thermal slug velocity, which provides additional insight into the transient heat transfer behavior observed during the test.

### 3.1. Waterfall Plots

**Figure 11:**
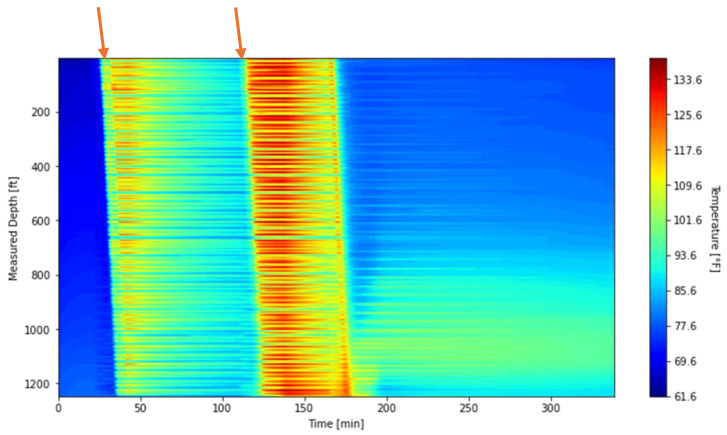
DTS waterfall plot for the entire circulation period.

Figure 11 presents the DTS waterfall plot along the well for the entire circulation test. The DTS measurements were recorded from 09:25:36 to 15:04:26. The plot clearly captures two thermal slugs, generated by separate hot-water injection events, which are indicated by the orange arrows.

As shown in Figure 11, the temperature profile is not smooth. With depth, the DTS temperature alters between local hot and cool spots. We attribute these local temperature variations to wellbore completion effects (relating to how the fiber optic cable was installed). On installation, the capillary tubing containing the fiber-optic cables was banded to the tubing approximately every 10 ft. This indicates that the measured temperature at each depth is strongly influenced by the fiber–tubing contact condition.

**Figure 12:**
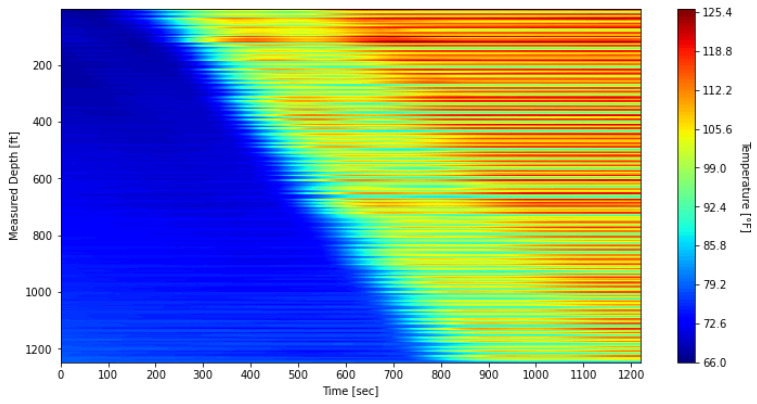
DTS waterfall plot during the first warm-up period, before processing.

**Figure 13:**
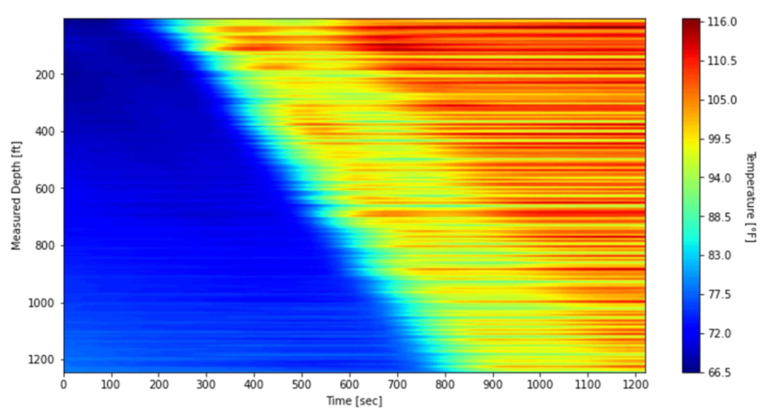
DTS waterfall plot during the first warm-up period, after processing.

**Figure 14:**
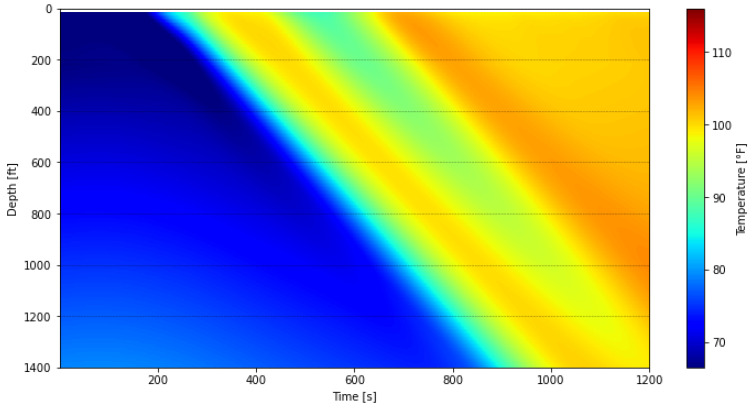
Model simulated waterfall plot during the first warm-up period.

To mitigate the impact of these banding effects on the subsequent thermal slug velocity extraction discussed in Section 3.2, the data was processed using a depth-averaging method applied to the first warm-up period. The unprocessed waterfall plot, covering DTS measurements from 09:47:59 to 10:08:20, is shown in Figure 12, while the processed results are presented in Figure 13. In this processing step, the original 1496 temperature measurement points were grouped into 187 sections, with each section representing the average of eight fiber-optic channels at each timestep. For comparison with these field-based waterfall plots, the simulated result is also presented in Figure 14.

The simulated plot reproduces the warm-up trend and shows a pattern similar to the DTS response in Figure 12 and Figure 13. The relatively lower temperatures in the model can be attributed to differences in measurement location. In the simulations, nitrogen temperatures are recorded at the midpoint of the annulus between the tubing and the inner casing, whereas the DTS system measures temperatures with fiber-optic cables housed in the capillary tubing that is banded to the outer surface of the tubing. This placement makes the DTS response more representative of the nitrogen temperature near the tubing outer diameter rather than at the annulus midpoint. Although the fiber is shielded by the capillary tubing and does not record the fluid temperature directly at that location, the proximity to the tubing wall likely causes the DTS measurements to reflect slightly higher temperatures than those predicted by the model. Consequently, the temperature rise in Figure 14 exhibits a lower trend than in DTS measurements and is not expected to completely coincide with them. Despite these differences, the overall consistency confirms that the numerical model captures the transient thermal behavior during the warm-up injection and supports its use for interpreting DTS data under similar test conditions.

### 3.2. Thermal Slug Velocities

Thermal slug velocity is a key parameter for interpreting transient heat transfer behavior, and its accurate determination from DTS field data is essential. Conventional approaches often estimate velocities arbitrarily by drawing lines through thermal signals on waterfall plots (e.g., Figure 11, where the slope of orange arrows indicates the roughly estimated velocity). In this study, a systematic method is introduced that uniquely determines the velocity from the field DTS data, particularly in the presence of banding effects.

**Figure 15:**
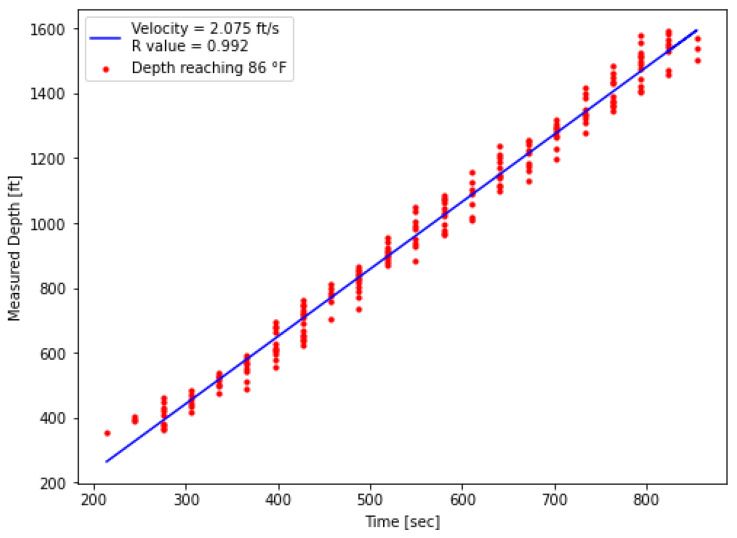
DTS measured thermal slug velocity at a threshold temperature of 86 °F.

For the extraction, the processed DTS data shown in Figure 13 was used. Prior to hot-water injection during the first warm-up period, the tubing fluid temperature was below 80 °F along the wellbore (Figure 9). A threshold temperature of 86 °F was defined to identify the arrival of the thermal front. At each measurement point, the time at which the temperature first exceeded this threshold was recorded and plotted on a time–depth diagram. A linear regression was then applied, and the slope of the fitted line provided the velocity estimate. As shown in Figure 15, the red dots represent threshold measurement points while the blue line indicates the fitted velocity which were estimated from the measured DTS data.

**Figure 16:**
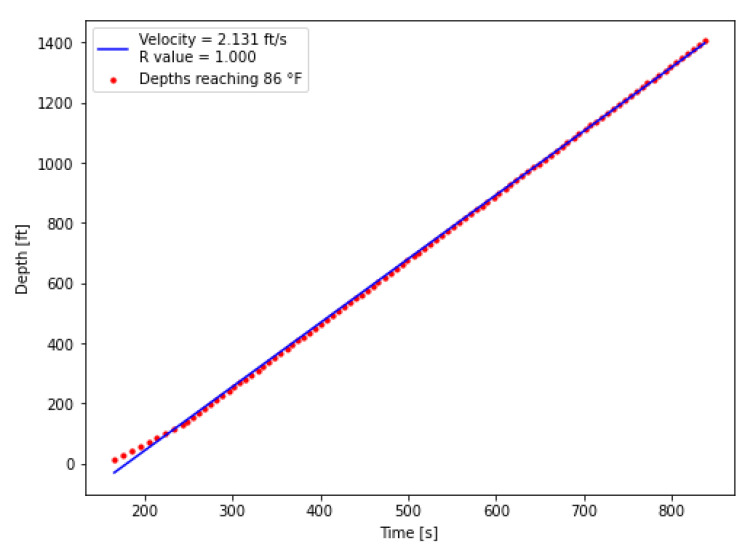
Model estimated thermal slug velocity at a threshold temperature of 86 °F.

In addition to completion effects, the 30 s DTS sampling interval also contributes to vertically aligned points on the diagram in Figure 15, as multiple depths may surpass the 86 °F threshold within the same time interval. These effects slightly reduce regression precision, but the effect is minor and remains within an acceptable range for velocity estimation. To enable direct comparison, the same extraction procedure was applied to the numerical model. The DTS- and model-derived velocities are summarized in Figure 15 and Figure 16.

From this analysis, the thermal slug velocities during the first warm-up injection were determined to be 2.075 ft/s from the DTS measurements and 2.131 ft/s from the numerical model. This close agreement not only demonstrates the reliability of the extraction procedure but also increases confidence in the model’s ability to capture the transient thermal response observed in the field. A comparison between the thermal slug velocity and the fluid velocity is provided in Table 7. The fluid velocity was calculated from the average flow rate reported in Table 6 and the tubing inside diameter. This comparison shows that the thermal slug velocity is consistently lower than the fluid velocity, reflecting the fact that heat transfer within the fluid is not instantaneous and therefore causes the thermal front to propagate more slowly than the fluid front.

## 4. Conclusions

In this study, we confirmed that the thermal slug velocity is lower than the fluid velocity. This finding provides new insight into heat transfer dynamics, showing that the thermal front propagates more slowly than the fluid front because heat transfer within the fluid is not instantaneous. The analysis highlighted strong completion effects, as the presence or absence of banding affected the DTS temperature profile and demonstrated the importance of accounting for completion design in data interpretation.

These findings were obtained by developing a transient wellbore heat transfer model and validating its predictions against DTS measurements. As part of this process, we introduced a threshold-temperature method to determine the thermal slug velocity in a non-arbitrary manner. When measurement points exceed the threshold at a given depth and time, they are recorded and plotted on time–depth diagrams. Linear regression is then applied to these points to calculate the velocity. This systematic approach removes the arbitrariness of conventional methods and yields consistent, reproducible velocity estimates. Model validation against DTS observations, including both waterfall plots and thermal slug velocities, showed close agreement, with velocity errors of less than 2.7%. In addition to its methodological advantages, the threshold method is practical and can be readily integrated into commercial DTS analysis platforms for automated velocity evaluation. Overall, the results demonstrate that the model reliably captures the dominant unsteady thermal behavior while retaining a simple framework and computational efficiency.

Looking ahead, future work will extend this approach to identify fracture locations from fiber-optic measurements and to quantify flow communication between injection and production wells in EGS. These developments will deepen the understanding of complex transient heat transfer in geothermal reservoirs and further enhance the role of fiber-optic sensing technology in geothermal development.

## Figures and Tables

**Table 1:** Planned test schedule for the circulation test.

Planned Activities
(1) Inject 50 bbls (7.95 m^3^) hot water at ~1 BPM (0.159 m^3^/min)
(2) 1 h shut-in while hot oil truck reloads with water and the well recovers temperature slightly
(3) Inject 50 bbls (7.95 m^3^) hot water at 1 BPM (0.159 m^3^/min)
(4) 1 h shut-in while hot oil truck reloads
(5) Inject 50 bbls (7.95 m^3^) cold water at 1 BPM (0.159 m^3^/min)

**Table 2:** Actual schedule for the circulation test.

Time	Operation
09:34 a.m.	Began water injection; surface pipe leak detected and repaired
09:48 a.m.	Injected hot water (~140 °F, ~60 °C)
10:08 a.m.	Shut-in
11:16 a.m.	Injected hot water (~158 °F, ~70 °C)
11:44 a.m.	Shut-in
12:12 p.m.	Injected cold water (~68 °F, ~20 °C)
12:41 p.m.	Shut down

**Table 3:** Simulation inputs.

Input	Value
Well length (TVD)	1405 ft (428.24 m)
Incremental length	14.05 ft (4.28 m)
Vertical grids	100
Radial grids	17
Time increments	1.0 s
Simulation period	20 min
Thermal diffusivity, $\hat{\alpha }$	0.04 ft^2^/hr (0.00372 m^2^/hr)
Dimensionless radius, $r_{D}$	2.000
Dimensionless time, $t_{D}$	0.05
Geometry Ratio, *a*	1.072

**Table 4:** Tubing, casing, and cement diameters and weight.

	ID		OD		Weight
	[in]	[cm]	[in]	[cm]	[lbm/ft]
Tubing	2.441	6.200	2.875	7.303	6.40
Inside Casing	4.892	12.43	5.500	13.97	17.0
Outside Casing	8.921	22.66	9.625	24.45	36.0
Cement	9.625	24.45	12.25	31.12	–

**Table 5:** Thermal conductivity, heat capacity, and density.

	Thermal Conductivity		Specific Heat		Density
	[Btu/hr-ft-°F]	[W/m-K]	[Btu/lbm-F]	[J/kg-K]	[lbm/ft^3^]
Water	0.32	0.55	1.00	4186.8	62.24
Tubing and Casing	25	43.24	0.12	502.42	–
Nitrogen	0.018	0.031	0.248	1038.3	1.510
Cement	0.5	0.86	0.37	1549.12	89.90
Formation	1.4	2.42	–	–	–

**Table 6:** Flow rate variation with time.

Time	Flow Rate [bbl/min]	Flow Rate [m^3^/min]
9:48:00–9:48:59	0.4088	0.0650
9:49:00–9:49:59	0.5975	0.0950
9:50:00–9:50:59	0.6604	0.1050
9:51:00–9:51:59	0.6667	0.1060
9:52:00–9:53:59	0.9938	0.1580
9:54:00–9:56:59	0.9919	0.1577
9:57:00–9:57:59	0.9938	0.1580
9:58:00–9:59:59	0.9969	0.1585
10:00:00–10:07:59	0.9963	0.1584

**Table 7:** Comparison of thermal and fluid velocities.

Thermal/Fluid Velocity	Velocity [ft/s]
Thermal slug velocity (DTS)	2.075
Thermal slug velocity (Model)	2.131
Fluid velocity	2.628

## Data Availability

All original findings presented in this study are contained within the article.

## Abbreviations

The following abbreviations are used in this manuscript:

DTS	Distributed temperature sensing
OTDR	Optical Time-Domain Reflectometry
EGS	Enhanced Geothermal Systems

## Appendix A

### Appendix A.1. Forced Convection

Forced convection arises when fluid motion is induced by external forces, such as pressure gradients encountered in oil production through a wellbore [29]. The associated heat transfer is commonly quantified through the convective heat transfer coefficient, which is typically estimated using empirical correlations. For turbulent flow in smooth tubes, one of the most widely applied correlations is the Dittus–Boelter equation [30], expressed as follows.

(A1) $$\mathrm{Nu}=0.023Re^{0.8}{\mathrm{Pr}}^{n}$$

(A2) 0.7 ≤ Pr ≤ 120, Re ≥ 104, L/D ≥ 60

(A3) Nu=htDk, Re=Dvρμ, Pr=cpμk

where *Nu*, *Re*, and *Pr* represent Nusselt, Reynolds, and Prandtl number, respectively. L/D is the length/diameter ratio of the tube. The exponent *n* of the Prandtl number is typically taken as 0.4 when the fluid is being heated, and 0.3 when it is being cooled. Using Equation (A1) through Equation (A3), the forced convection heat transfer coefficient $h_{t}$ is calculated.

### Appendix A.2. Natural Convection

In the absence of fluid flow, natural convection governs heat transfer between a fluid and a surface when a temperature difference exists, driven by buoyancy forces arising from density gradients [29]. For instance, in the transfer of heat from the tubing outer wall to the casing inner wall, conduction is not the dominant mechanism; rather, the process is more accurately described by a convective heat transfer model with an appropriate heat transfer coefficient. Research on natural convection in vertical annular geometries remains limited. A notable contribution is the correlation proposed by Dropkin and Sommerscales for fluids confined between two vertical plates [24], which has since been reformulated for cylindrical geometries. The resulting expression for the natural convection heat transfer coefficient, *h_a_*, is given as follows:

(A4) $$h_{a}\;=\;\frac{0.049{\left(\mathrm{GrPr}\right)}^{0.333}{\mathrm{Pr}}^{0.074}k_{a}}{r_{\mathrm{to}}\ln(r_{\mathrm{ci}}/r_{\mathrm{to}})}$$

(A5) $$\mathrm{Gr}=\frac{{\left(r_{\mathrm{ci}}-r_{\mathrm{to}}\right)}^{3}g\rho_{a}^{2}\beta (T_{\mathrm{to}}-T_{\mathrm{ci}})}{\mu_{a}^{2}}$$

where *Gr* is Grashof number and reflects the extent of motion of the annular fluid owing to natural convection [27]. $r_{\mathrm{to}}$ and $r_{\mathrm{ci}}$ represent the tubing outer radius and casing inner radius, and $T_{\mathrm{to}}$ and $T_{\mathrm{ci}}$ are the temperatures at those points. Using Equation (A3) through Equation (A5), the natural convection heat transfer coefficient $h_{a}$ is calculated.
